# Evaluation of the effects of meteorological factors on COVID-19 prevalence by the distributed lag nonlinear model

**DOI:** 10.1186/s12967-022-03371-1

**Published:** 2022-04-11

**Authors:** Hongjing Ai, Rongfang Nie, Xiaosheng Wang

**Affiliations:** 1 Biomedical Informatics Research Lab, School of Basic Medicine and Clinical Pharmacy, Jiangning District, China Pharmaceutical University, No. 639 Longmian Avenue, Nanjing, 211198 Jiangsu China; 2 Big Data Research Institute, China Pharmaceutical University, Nanjing, 211198 China

**Keywords:** COVID-19, Meteorological factors, Distributed lag nonlinear model, Relative risk

## Abstract

**Background:**

Although numerous studies have explored the impact of meteorological factors on the epidemic of COVID-19, their relationship remains controversial and needs to be clarified.

**Methods:**

We assessed the risk effect of various meteorological factors on COVID-19 infection using the distributed lag nonlinear model, based on related data from July 1, 2020, to June 30, 2021, in eight countries, including Portugal, Greece, Egypt, South Africa, Paraguay, Uruguay, South Korea, and Japan, which are in Europe, Africa, South America, and Asia, respectively. We also explored associations between COVID-19 prevalence and individual meteorological factors by the Spearman’s rank correlation test.

**Results:**

There were significant non-linear relationships between both temperature and relative humidity and COVID-19 prevalence. In the countries located in the Northern Hemisphere with similar latitudes, the risk of COVID-19 infection was the highest at temperature below 5 ℃. In the countries located in the Southern Hemisphere with similar latitudes, their highest infection risk occurred at around 15 ℃. Nevertheless, in most countries, high temperature showed no significant association with reduced risk of COVID-19 infection. The effect pattern of relative humidity on COVID-19 depended on the range of its variation in countries. Overall, low relative humidity was correlated with increased risk of COVID-19 infection, while the high risk of infection at extremely high relative humidity could occur in some countries. In addition, relative humidity had a longer lag effect on COVID-19 than temperature.

**Conclusions:**

The effects of meteorological factors on COVID-19 prevalence are nonlinear and hysteretic. Although low temperature and relative humidity may lower the risk of COVID-19, high temperature or relative humidity could also be associated with a high prevalence of COVID-19 in some regions.

## Introduction

The coronavirus disease 2019 (COVID-19) pandemic has caused more than 470 million cases and six million deaths across the world as of March 28, 2022 [1]. It still constitutes an extraordinary event and continues to affect the human health around the world [2]. Like other emerging infectious diseases, COVID-19 occurrence and spread are affected by a variety of factors, including external politics, economy, culture, climate and ecological conditions [3] as well as internal human immunity. However, compared to most emerging infectious diseases, COVID-19 is more infectious and more challenging to be contained.

Usually, the spread of respiratory infectious diseases is sensitive to seasonal changes [4]. Nevertheless, the relationship between meteorological factors and COVID-19 remains insufficiently definite, although plentiful studies on this topic have been published. For example, Lim et al. [5] found that the duration of sunshine and ozone level were positively correlated with the number of COVID-19 cases in two regions of the Republic of Korea, while temperature variables showed contradictory results. Liu et al. [6] used generalized linear model combined with meta-analysis to demonstrate that low temperature, mild diurnal temperature difference and low humidity might be conducive to COVID-19 transmission. Cacho et al. [7] revealed that ultraviolet (UV) radiation and temperature played a critical role in the spread of COVID-19 by establishing a linear regression model. Daneshvar et al. [8] made a comparative analysis between United Arab Emirates and Switzerland and revealed that the climate effects on the COVID-19 varied in different countries. Bilal et al. [9] proved that the PM2.5, environmental quality index and precipitation were important factors in the transmission of COVID-19 in the United States.

To explore the effects of meteorological factors on the COVID-19 epidemic, we explored the COVID-19 epidemiological data and meteorological data from eight countries, including Portugal, Greece, Egypt, South Africa, Paraguay, Uruguay, South Korea, and Japan. These countries are located on four continents and satisfy the following criteria: variable climate conditions and small country area. Generally, the effects of climate factors on health are nonlinear [10] and hysteretic [11]. Also, the COVID-19 prevalence is associated with human activities, such as the policy responses and public behaviors [12]. Because prior studies of the relationships between meteorological factors and COVID-19 prevalence often ignored confounding factors, we used the distributed lag nonlinear model (DLNM) to explore their relationships by including various confounding variables in the model, based on the data from July 1, 2020, to June 30, 2021, in the eight countries. We also explored associations between COVID-19 prevalence and individual meteorological factors by the Spearman’s rank correlation test in these countries.

## Methods

### Data collection and processing

**Fig. 1:**
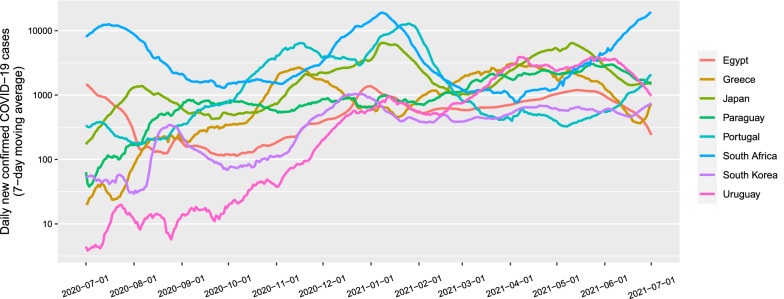
7-day moving average of the daily new confirmed COVID-19 cases. For better visualization, the logarithmic Y-axis is used because the large order-of-magnitude differences in numbers of daily new confirmed COVID-19 cases among the countries

We collected data from July 1, 2020 to June 30, 2021. The number of daily new confirmed cases (DNCCs) of COVID-19 were obtained from the Center for Systems Science and Engineering (CSSE) of Johns Hopkins University [13]. Meteorological data (temperature, precipitation, relative humidity, UV index, NO_2_ total column, and UV aerosol index) were obtained from the Geospatial Interactive Online Visualization and Analysis Infrastructure (Giovanni) of National Aeronautics and Space Administration (NASA) [14]. The data of government response stringency index, COVID-19 vaccination, Google mobility trends and face coverings policies were obtained from the Our World in Data [15]. Because the original daily numbers of new confirmed cases showed large variability, we took their 7-day moving average to reduce the effects of random fluctuations and the weekly effect (Fig. 1). We used the processed data for subsequent analyses.

### Spearman correlation analysis

Because the epidemiological data and meteorological data did not follow normal distribution (Shapiro–Wilk test, *P* < 0.05), we analyzed Spearman correlations between numbers of DNCCs of COVID-19 and values of meteorological factors and between different meteorological factors. Considering that the median incubation period for COVID-19 is around 7 days [16, 17], we analyzed the correlation between meteorological factors and numbers of DNCCs of COVID-19 with a 7-day lag.

### Distributed lag nonlinear model (DLNM)

The DLNM model is based on the concept of cross-basis, a bi-dimensional space of functions describing simultaneously the shape of the relationship along both the space of the predictor and the lag dimension of its occurrence [18]. DLNM selects appropriate basis functions for two dimensions to generate the cross-basis by taking tensor product and transforms the original variables to obtain new values included in the model [18]. DLNM is defined as follows:

$$\text{log}\left(E\left({Y}_{t}\right)\right)=\alpha +cb\left({x}_{t};\eta \right)+\sum_{k=1}^{K}{\gamma }_{k}{\mu }_{tk}$$

where ${Y}_{t}$ is the time series responding variable on day $t$, which follows a family of exponential distributions; $\alpha$ is the intercept and $cb$ denotes the cross-basis function of predictor variable $x$; ${\gamma }_{k}$ represents the confounding variable; $\eta$ and ${\mu }_{tk}$ denote the parameter vector and the coefficient, respectively.

Because the numbers of DNCCs are over dispersed, we assumed that they followed a quasi-Poisson distribution. Because of the correlation between meteorological factors, we incorporated variables of individual meteorological factors into the DLNM. We set the maximum lag period as 21 days, based on previous estimates of the incubation period for COVID-19 [19, 20]. We set the variable *Time* in the model to adjust for long-term trends. The model included several confounding variables, including cumulative rates of vaccination (*VAC*), government response stringency index (*GRSI*), Google mobility trends (*GMT*), and face coverings policies (*FCP*). *GMT* indicates the number of visitors to specific categories of location. We used the logarithmic conversion of DNCCs on day *t−1* to control for autocorrelation [21]. To avoid overfitting, the degree of freedom (*df*) for the natural cubic spline functions (*ns*) of both exposure dimension, lag dimension, and variable $Time$ were limited to less than 6, 6, and 10, respectively. The modified Akaike Information Criterion (AIC) was used to determine the *df* [22, 23]. The median value of meteorological data was used as a reference to estimate, and the relative risk (RR) with a 95% confidence interval (CI) was used to evaluate the effect. The model was finally established as follows:

$$\text{log}\left(E\left({Y}_{t}\right)\right)=\, \alpha +cb\left({x}_{t};\eta \right)+ns\left(time,df\right)+VAC+GRSI+GMT+FCP+\text{log} ({Y}_{t-1})$$

## Results

### Descriptive statistics

The differences in meteorological data among countries are associated with geographical conditions. The mean values of meteorological data in the eight countries are shown in Table 1.

**Table 1:** Average of meteorological data in different countries

Country	Temperature (℃)	Precipitation (mm)	Relative humidity (%)	Ultraviolet index	NO_2_ total column (1/cm^2^)	Ultraviolet aerosol index
Portugal	18.42	2.62	53.31	5.50	3.99E+15	1.38
Greece	18.02	3.00	65.77	5.17	3.80E+15	1.48
Egypt	27.71	0.08	26.81	8.62	3.37E+15	1.53
South Africa	22.81	1.57	39.80	8.13	3.74E+15	0.94
Paraguay	28.05	3.37	46.90	8.21	3.17E+15	0.96
Uruguay	20.78	4.06	58.41	6.78	3.47E+15	0.95
South Korea	16.04	3.88	67.42	4.39	7.28E+15	1.38
Japan	15.34	5.20	71.57	4.59	5.20E+15	1.08

### Correlation analysis

**Fig. 2:**
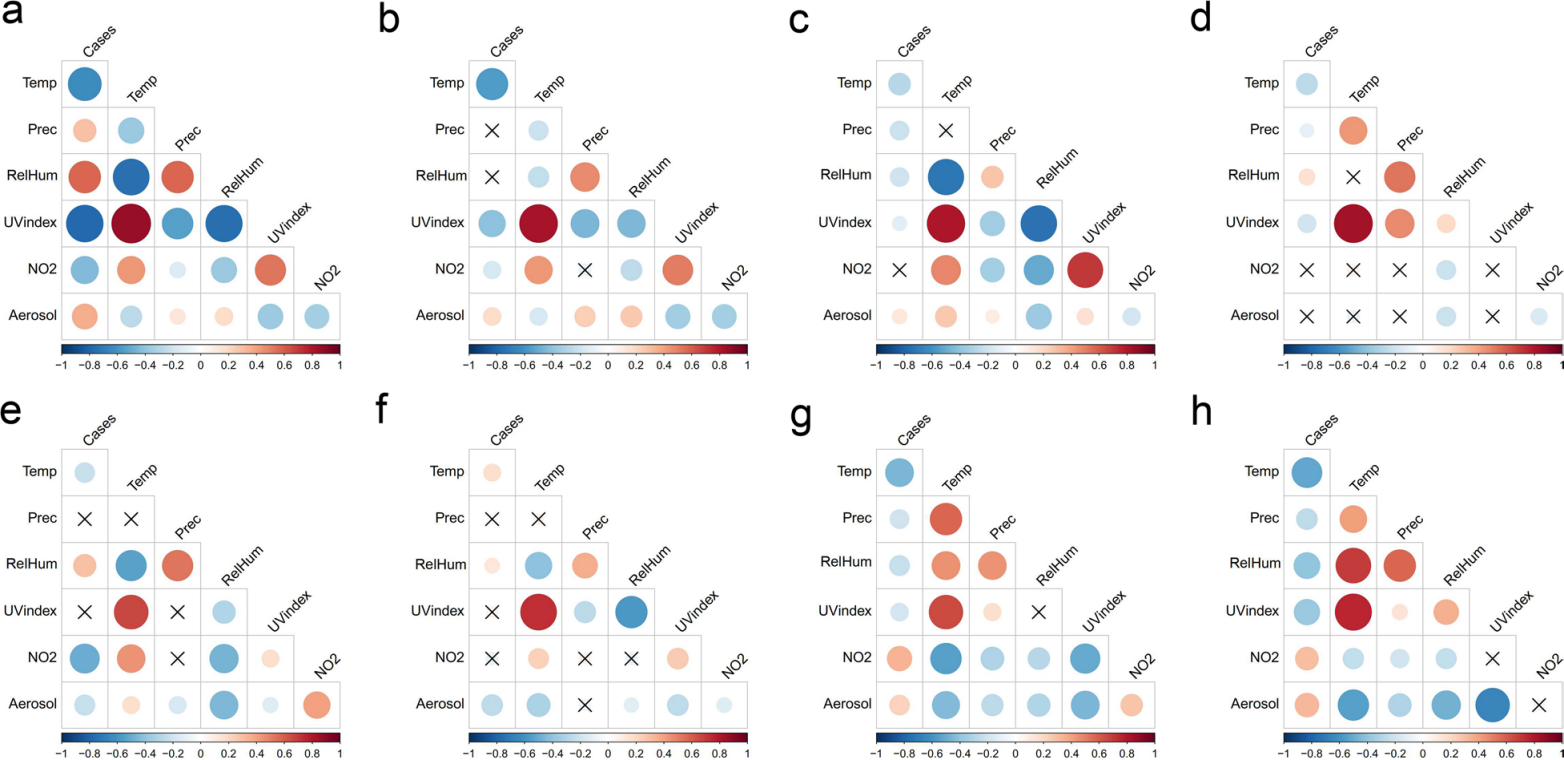
Pairwise Spearman correlations between the number of daily new confirmed COVID-19 cases and meteorological factors and between different meteorological factors in nine countries. The red and blue represent positive and negative correlations, respectively. The color gradient and circle size are proportional to correlation coefficient, and the cross indicates that the statistical test is not significant (*P* ≥ 0.05). **a** Portugal; **b** Greece; **c** Egypt; **d** South Africa; **e** Paraguay; **f** Uruguay; **g** South Korea; **h** Japan. Cases: daily new confirmed COVID-19 cases; Temp: temperature; Prec: precipitation; RelHum: relative humidity; UV index: ultraviolet index; NO2: NO_2_ total column; Aerosol: ultraviolet aerosol index

The Spearman correlations between the number of DNCCs of COVID-19 and meteorological factors in each of the eight countries are shown in Fig. 2 and Table 2. Notably, there were discrepancies in the correlations among different countries. For example, temperature and the number of COVID-19 cases had a significant negative correlation in seven countries (Portugal, Greece, Egypt, South Africa, Paraguay, South Korea, and Japan), while they had a significant positive correlation in Uruguay (*P* < 0.05). UV index was significantly and negatively correlated with the number of COVID-19 cases in six countries (Portugal, Greece, Egypt, South Africa, South Korea, and Japan). Relative humidity and the number of COVID-19 cases had a significant positive correlation in four countries (Portugal, South Africa, Paraguay, and Uruguay), while they had a significant negative correlation in Egypt, South Korea, and Japan. The correlation between UV aerosol index and COVID-19 was significant and positive in five countries (Portugal, Greece, Egypt, South Korea, and Japan), while it was negative in Paraguay and Uruguay. The correlations of precipitation and NO_2_ with COVID-19 were dependent on countries, being positive, negative, or not significant. In addition, there was a strong positive correlation (*P* < 0.05; *ρ* > 0.5) between temperature and UV index in 7 countries (Portugal, Greece, Egypt, South Africa, Paraguay, Uruguay and South Korea) and between relative humidity and precipitation in 4 countries (South Africa, Paraguay, Uruguay and Japan).

**Table 2:** Spearman correlations between the numbers of DNCCs of COVID-19 and meteorological factors

Meteorological factor	Country							
	Portugal	Greece	Egypt	South Africa	Paraguay	Uruguay	South Korea	Japan
Temperature	− 0.63*	− 0.57*	− 0.28*	− 0.26*	− 0.23*	0.17*	− 0.45*	− 0.52*
Precipitation	0.3*	0.04	− 0.21*	− 0.11*	− 0.01	− 0.05	− 0.21*	− 0.25*
Relative humidity	0.58*	− 0.05	− 0.21*	0.15*	0.29*	0.13*	− 0.24*	− 0.39*
UV index	− 0.77*	− 0.41*	− 0.12*	− 0.19*	− 0.01	0.07	− 0.18*	− 0.38*
NO_2_ total column	− 0.43*	− 0.18*	0.02	0.02	− 0.49*	− 0.09	0.35*	0.31*
UV aerosol index	0.36*	0.19*	0.13*	0.02	− 0.24*	− 0.26*	0.23*	0.33*

*P < 0.05

### Analysis by DLNM

**Fig. 3:**
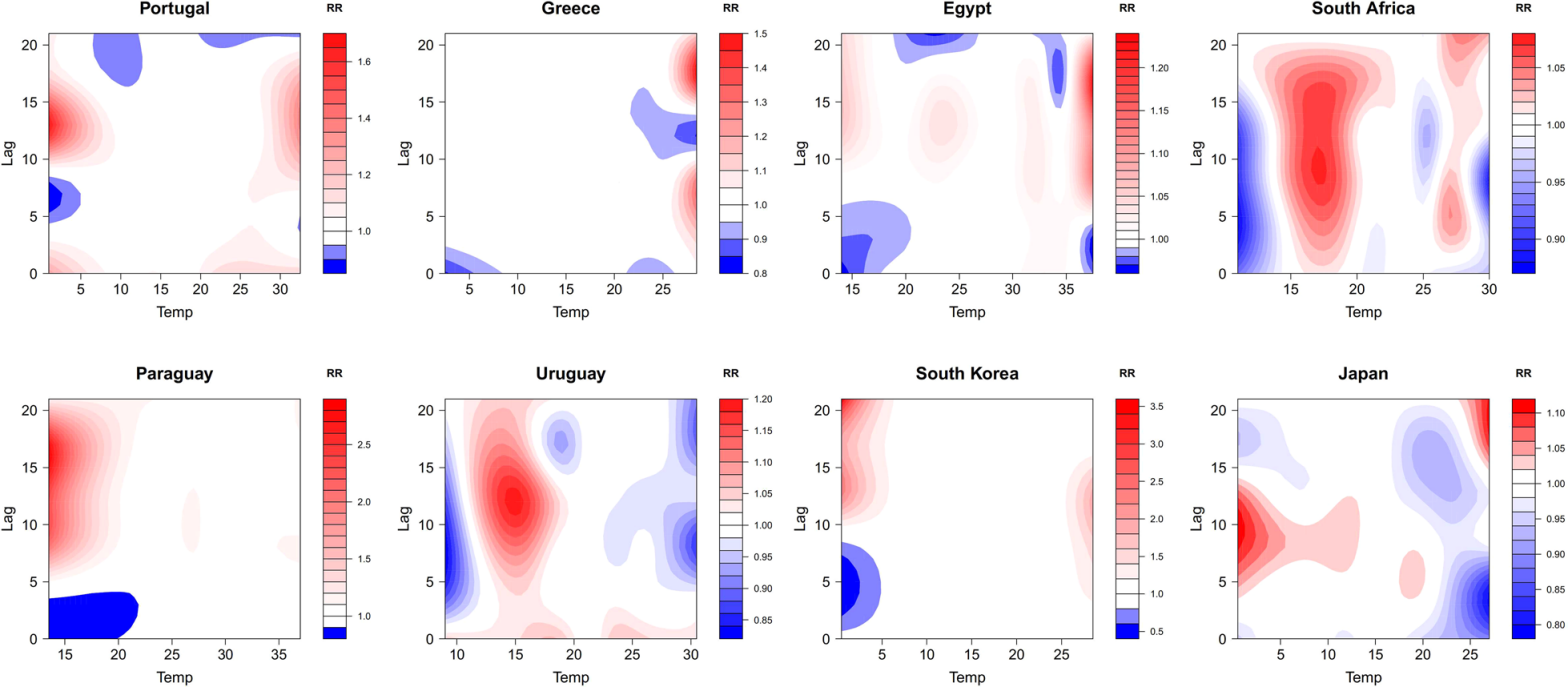
Contour plots of the relative risk (RR) along temperature and lag time on COVID-19 infection. The X-axis represents the meteorological value and the Y-axis represents lag days ranging from 0 to 21 days. The RR was determined based on the median value of meteorological data. The red and blue indicate RR greater than 1 and less than 1, respectively

Based on the results of correlation analyses, we included two meteorological factors with relatively consistent results (temperature and UV index) and one with different results among countries (relative humidity) in the DLNM for effect analysis. Figure 3 shows the contour plots of the RR along temperature and lag time on the number of DNCCs of COVID-19. In three countries (Portugal, South Korea, and Japan), the risk for COVID-19 infection at temperature < 5 °C was significantly higher than that at high temperature (> 25 °C). Portugal had the highest risk of infection at 1 °C with a lag of 13 days (RR = 1.666; 95% CI 1.280 ~ 2.170). South Korea and Japan had the highest RR when the temperature was 0.5 °C with a lag of 21 days (RR = 3.510; 95% CI 1.888 ~ 6.527) and 10 days (RR = 1.115; 95% CI 1.067 ~ 1.165), respectively. Interestingly, in all the three countries, there was also a high risk of infection when the temperature was above 25 °C with a certain lag time (13 days, 10 days, and 21 days in Portugal, South Korea, and Japan). In three Southern Hemisphere countries (South Africa, Paraguay, and Uruguay), the highest RR occurred at around 15 ℃ with a lag of long time (0–20 days, 5–21 days, and 0-21 days in South Africa, Paraguay, and Uruguay). The RR in South Africa reached its maximum value of 1.073 (95% CI 1.036 ~ 1.111) at 17 ℃ with a lag of 9 days; the RR in Paraguay reached its maximum value of 2.841 (95% CI 1.891 ~ 4.269) at 13.5 ℃ with a lag of 16 days; the RR in Uruguay reached its maximum value of 1.191 (95% CI 1.137 ~ 1.248) at 15 ℃ with a lag of 12 days. However, in Greece and Egypt, the risk for COVID-19 infection at high temperature was higher than that at low temperature. Greece had the highest RR at temperature of 28.5 ℃ (RR = 1.497; 95% CI 1.275 ~ 1.759), and Egypt had the highest RR at temperature of 37.5 ℃ (RR = 1.234; 95% CI 1.175 ~ 1.296). Although the lag effect of temperature varied from country to country, the lag time with a higher risk of infection was likely longer than the average incubation period (7 days).

**Fig. 4:**
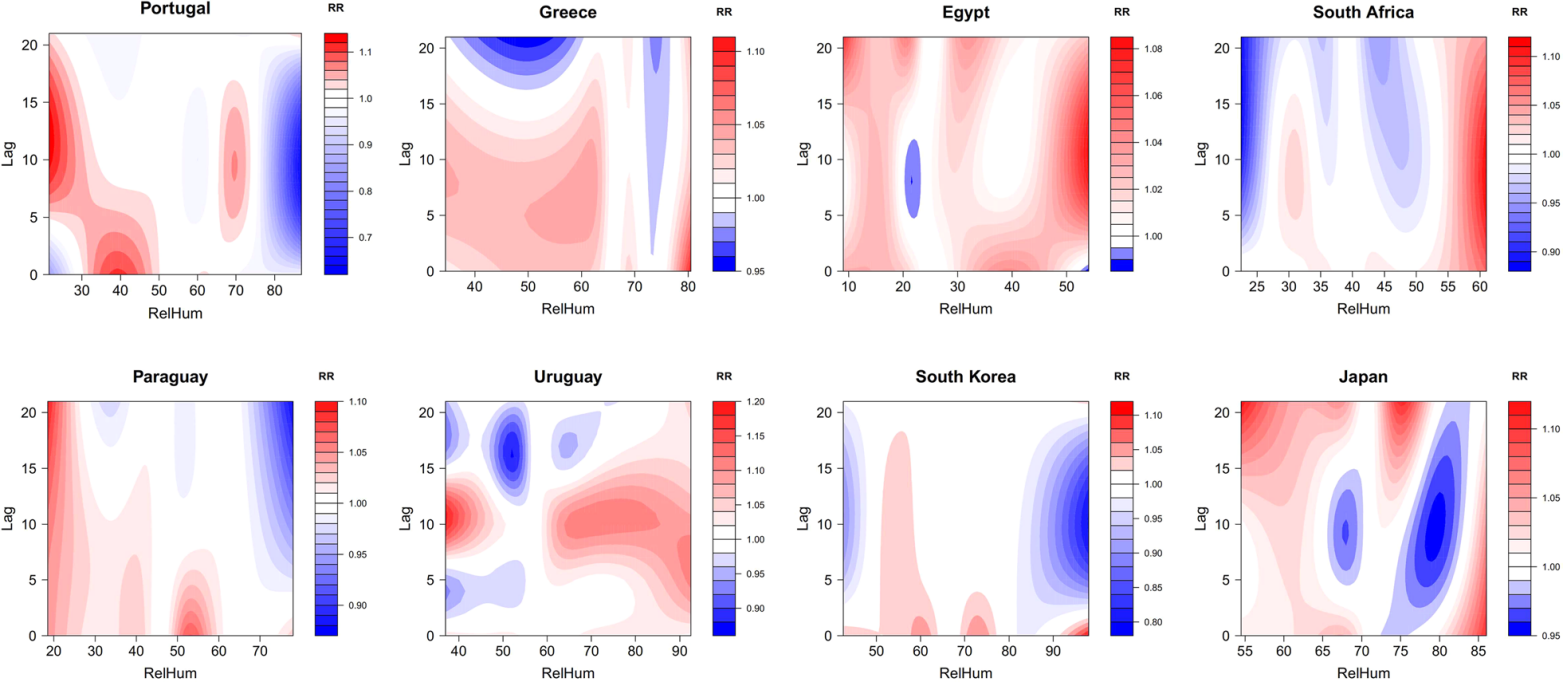
Contour plots of the RR along relative humidity and lag time on COVID-19 infection

Figure 4 presents the contour plots of the RR along relative humidity and lag time on the number of DNCCs of COVID-19. Egypt and South Africa had year-round low relative humidity. The highest risk in both countries occurred at their high relative humidity (54% relative humidity for Egypt and 61% relative humidity for South Africa). In particular, the RR reached its peak of 1.111 (95% CI 1.077 ~ 1.147) when the relative humidity was 61% and 9 days lagged in South Africa. In countries with a large range of relative humidity, lower relative humidity was associated with a higher RR of COVID-19 infection, and the lag effect generally lasted for a long time. In Portugal, the RR was the highest within lag of between 5 and 21 days at relative humidity < 30%; the RR in Paraguay was the highest within lag of between 0 and 21 days at relative humidity < 25%. In the other countries, although there was a higher risk of infection at low relative humidity, the high RR at high relative humidity could occur. For example, Greece had the highest risk of infection at relative humidity up to 80.5% (RR = 1.102; 95% CI 1.008 ~ 1.206); Uruguay had a high risk of infection at relative humidity > 60%. Moreover, compared to temperature, the lag effect of relative humidity on COVID-19 lasted for a longer period (median period: 20-day period versus 14-day period).

**Fig. 5:**
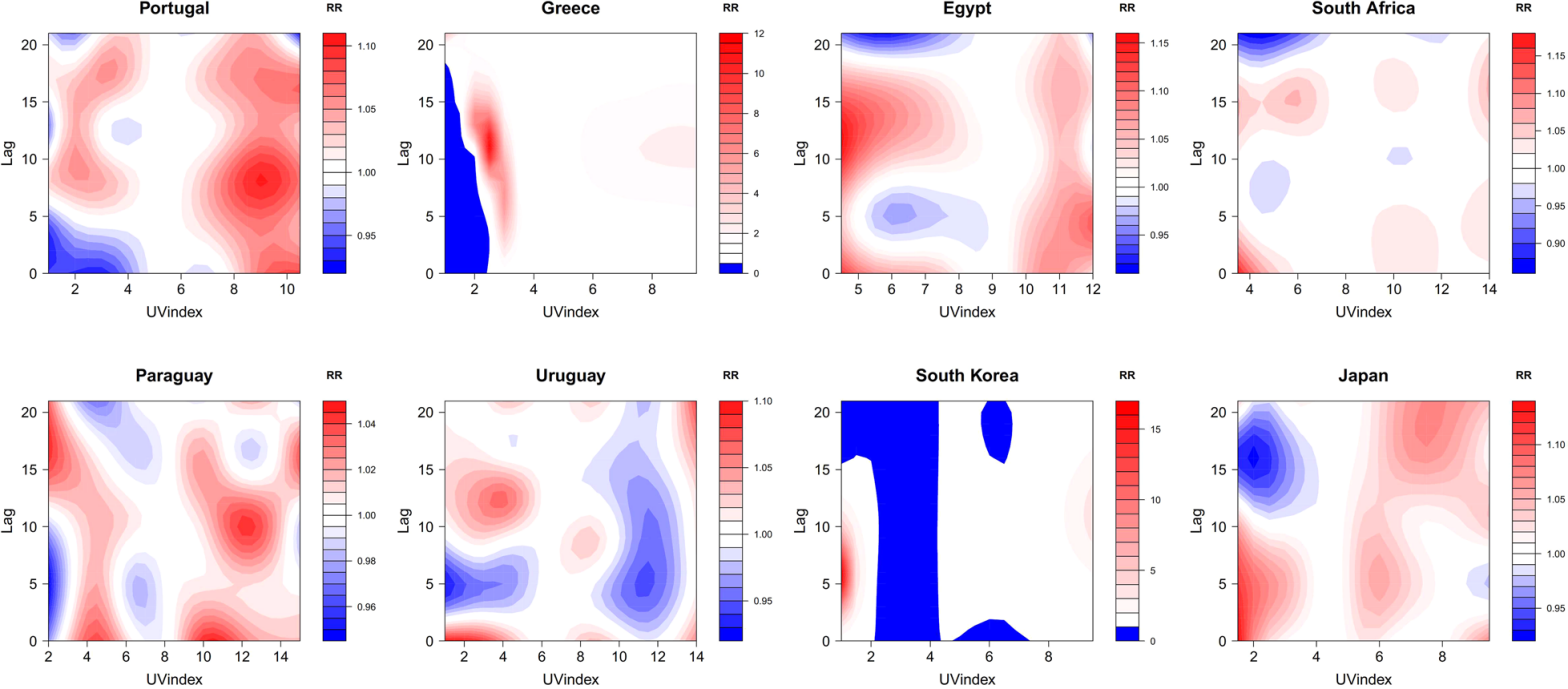
Contour plots of the RR along UV index and lag time on COVID-19 infection

The effect of UV index on COVID-19 infection varied across the countries, with the high RR was distributed in various regions of UV index values (Fig. 5). The contour plots show the characteristics of multiple centers of high RR. Portugal had the highest risk of infection when its UV index was 7 (RR = 1.164; 95% CI 1.112 ~ 1.218) with a lag of 5 days. Nevertheless, there were several centers of high infection in the regions of low UV index and high UV index. Similar nonlinear relationships were observed in Egypt, South Africa, Paraguay, Uruguay, and Japan.

## Discussion

The impact of meteorological factors on the epidemic of COVID-19 remains controversial. Most of previous studies reported that cold and dry climate conditions were conducive to the transmission of COVID-19 [24–26], while some showed that high temperature could not inhibit the transmission of COVID-19 [27] or that there was no significant correlation between temperature and COVID-19 infection [28, 29]. There are several reasons to explain these contradictory results. First, different research subjects may lead to different results, such as regional studies versus global studies. Second, many previous studies failed to cover all meteorological conditions. In addition, different research methods may lead to different results [27]. In this study, we aimed to capture common patterns or discrepancies of the relationship between meteorological factors and the COVID-19 epidemic among individual countries. A climate-dependent epidemic model showed that meteorological variables were unlikely to be dominant transmission risk factors in the early stages of the COVID-19 pandemic due to the high population susceptibility [30]. Besides, in order to minimize the influences of the variation of SARS-CoV-2 [31] and to obtain the maximum range of meteorological data, we analyzed data from July 1, 2020, to June 30, 2021, in eight countries from four continents. Spearman’s correlation analysis showed that temperature and UV index were negatively correlated with COVID-19 prevalence in 7 and 8 countries, respectively. The correlation between relative humidity and COVID-19 prevalence showed positive correlation in 4 countries and negative correlation in 3 countries. Thus, we included the three meteorological factors (temperature, relative humidity, and UV index) in the DLNM for risk analysis, respectively. Our results showed a significant non-linear relationship between temperature and COVID-19 prevalence. Portugal, Greece, South Korea, and Japan are in the Northern Hemisphere and have similar latitudes. Three of them (Portugal, South Korea, and Japan) had a higher risk of COVID-19 infection at low temperature (< 5 ℃), and all the four countries also had a higher risk of infection at high temperature (> 25 ℃) with certain lag days. South Africa, Paraguay, and Uruguay are all in the Southern Hemisphere with similar latitudes, and their highest risk of infection occurred at around 15 ℃. In addition, there was a significant lag effect of temperature on COVID-19 prevalence, with the lag time for the occurrence of the highest RR longer than the estimated mean incubation period of COVID-19 in all the eight countries. We also proved that the pattern of risk effects of relative humidity on COVID-19 infection largely depended on the variation range of year-round relative humidity in countries. Lower relative humidity was associated with higher COVID-19 prevalence in countries with a wide range of relative humidity, while the relative risk of COVID-19 infection in high relative humidity could be high in countries with overall high relative humidity. Moreover, the lag effect of relative humidity generally lasts for a long time. Nevertheless, the non-linear effects of UV index on COVID-19 prevalence were polycentric and varied across countries. The potential reason could be that our UV index data only represented the outdoor air conditions, while epidemiological tracing reports indicated that the infection rate indoors was much higher than that outdoors [32].

Since the first launch of COVID-19 vaccines in December 2020, around 65% of the world population has received at least one dose of a COVID-19 vaccine. The protective effect of vaccines can slow down the COVID-19 transmission [33, 34]. Besides, the adjustment of government prevention and control policies has played a crucial role in containing the spread of COVID-19 [35]. In addition, wearing masks and social distancing have a direct effect on controlling the spread of COVID-19 [36, 37]. Therefore, our model included the related variables, such as cumulative vaccination rate, government response stringency index, face coverings policies, and Google mobility trends. Although we controlled the above factors, there are still some limitations. First, the number of DNCCs of COVID-19 notified by the official authorities may be omitted. Second, meteorological data were measured by remote sensing, which represented the average meteorological value of a country. The larger the country area, the less likely the meteorological data are accurate. Finally, we only included one meteorological factor at a time in constructing the DLNM, while the real exposure condition was a combination of various meteorological factors. As a result, the dominant meteorological factor cannot be identified.

## Conclusions

The effects of meteorological factors on COVID-19 transmission are nonlinear and hysteretic. Lower temperature and lower relative humidity were associated with a higher risk of COVID-19 infection. However, the non-linear effects of meteorological factors on COVID-19 transmission should not be ignored. In some countries, high temperature or high relative humidity may also enhance the risk of COVID-19 infection. It is necessary to consider the meteorological factors into the risk assessment of COVID-19 transmission, but the impact of meteorological factors on the transmission of COVID-19 may be weaker compared with other factors, such as virus mutations, vaccination, social distance, and government prevention and control policies.

## Data Availability

The datasets analyzed during the current study are available from the corresponding author on reasonable request.

## Abbreviations

COVID-19: Coronavirus disease 2019
DLNM: Distributed lag nonlinear model
DNCCs: Daily new confirmed cases
CSSE: Center for Systems Science and Engineering
UV: Ultraviolet
Giovanni: Geospatial Interactive Online Visualization and Analysis Infrastructure
NASA: National Aeronautics and Space Administration
*VAC*: Vaccination
*GRSI*: Government response stringency index
*GMT*: Google mobility trends
*FCP*: Face coverings policies
*df*: Degree of freedom
AIC: Akaike Information Criterion
RR: Relative risk
CI: Confidence interval
Temp: Temperature (℃)
Prec: Precipitation (mm)
RelHum: Relative humidity (%)
NO2: NO_2_ total column (1/cm^2^)
Aerosol: Ultraviolet aerosol index
